# Ascophyllan Purified from *Ascophyllum nodosum* Induces Th1 and Tc1 Immune Responses by Promoting Dendritic Cell Maturation

**DOI:** 10.3390/md12074148

**Published:** 2014-07-14

**Authors:** Wei Zhang, Jiang-Yuan Du, Zedong Jiang, Takasi Okimura, Tatsuya Oda, Qing Yu, Jun-O Jin

**Affiliations:** 1 Shanghai Public Health Clinical Center, Shanghai Medical College, Fudan University, Shanghai 201508, China; E-Mails: weiwei061215@126.com (W.Z.); dujiangyuan@shaphc.org (J.-Y.D.); 2 Division of Biochemistry, Faculty of Fisheries, Nagasaki University, 1-14 Bunkyo-machi, Nagasaki 852-8521, Japan; E-Mail: zdjiang1982@hotmail.com; 3 Research and Development Division, Hayashikane Sangyo Co., Ltd., Shimonoseki, Yamaguchi 750-8608, Japan; E-Mail: tokimura@hayashikane.co.jp; 4 Graduate School of Science and Technology, Nagasaki University, 1-14 Bunkyo-machi, Nagasaki 852-8521, Japan; E-Mail: t-oda@nagasaki-u.ac.jp; 5 Department of Immunology and Infectious Diseases, The Forsyth Institute, 245 First Street, Cambridge, MA 02142, USA; E-Mail: qyu@forsyth.org

**Keywords:** ascophyllan, dendritic cells, DC maturation, Th1 cells, Tc1 cells

## Abstract

Marine-derived sulfated polysaccharides have been shown to possess certain anti-virus, anti-tumor, anti-inflammatory and anti-coagulant activities. However, the *in vivo* immunomodulatory effects of marine-derived pure compounds have been less well characterized. In this study, we investigated the effect of ascophyllan, a sulfated polysaccharide purified from *Ascophyllum nodosum*, on the maturation of mouse dendritic cells (DCs) *in vitro* and *in vivo*. Ascophyllan induced up-regulation of co-stimulatory molecules and production of pro-inflammatory cytokines in bone marrow-derived DCs (BMDCs). Moreover, *in vivo* administration of ascophyllan promotes up-regulation of CD40, CD80, CD86, MHC class I and MHC class II and production of IL-6, IL-12 and TNF-α in spleen cDCs. Interestingly, ascophyllan induced a higher degree of co-stimulatory molecule up-regulation and pro-inflammatory cytokine production than fucoidan, a marine-derived polysaccharide with well-defined effect for promoting DC maturation. Ascophyllan also promoted the generation of IFN-γ-producing Th1 and Tc1 cells in the presence of DCs in an IL-12-dependent manner. Finally, myeloid differentiation primary response 88 (MyD88) signaling pathway was essential for DC maturation induced by ascophyllan. Taken together, these results demonstrate that ascophyllan induces DC maturation, and consequently enhances Th1 and Tc1 responses *in vivo*. This knowledge could facilitate the development of novel therapeutic strategies to combat infectious diseases and cancer.

## 1. Introduction

One of the new research trends in the development of immunomodulatory agents is to search for candidates among natural products, because they have relatively low or tolerable toxicity in clinical applications [1,2]. After decades of extensive studies, some marine-derived pure compounds with strong immunomodulatory activities have been identified, mostly through *in vitro* studies. The *in vivo* immunomodulation effects of marine-derived pure compounds have been less well investigated. Ascophyllan is a complex and heterogeneous sulfated polysaccharide, which is extracted from brown alga *Ascophyllum nodosum* (*A. nodosum*) [3]. The characteristic monosaccharide composition of ascophyllan is obviously distinguishable from those of fucoidan, which contains fucose as a main sugar component with sulfate groups [4]. A recent study has demonstrated that ascophyllan induces much higher levels of nitric oxide (NO) production from mouse macrophage cell line RAW264.7 than fucoidan [5]. In an *in vitro* study, ascophyllan was shown to promote secretion of tumor necrosis factor-α (TNF-α) and granulocyte colony-stimulating factor (G-CSF) from RAW264.7 cells [3]. Moreover, systemic administration of ascophyllan enhances splenic natural killer (NK) cell activity against murine lymphoma cell line YAC-1 [6]. In contrast to its effects on macrophages and NK cells, the effects of ascophyllan on DCs, especially spleen DCs, are largely uncharacterized.

Dendritic cells (DCs) play a key role in initiating and controlling the magnitude and the quality of adaptive immune responses [7,8]. Upon exposure to microbial stimuli, DCs undergo a maturation process characterized by increased expression of co-stimulatory molecules, production of pro-inflammatory cytokines and presentation of antigens (Ags) to T cells [7,8,9]. Different subsets of DCs show different specialized functions. CD8α^+^ conventional DCs (cDCs) have a selective ability to cross-present exogenous Ags though MHC class I [9,10,11]. This function is crucial for the generation of cytotoxic T cells against virus and nuclear Ags from necrotic cells [11]. In contrast, the extracellular antigens are captured and moved to the endosome/lysosomes in CD8α^−^ cDCs, which then are degraded to antigenic peptides and complexed with MHC class II molecules to be recognized by CD4 T cells [12,13]. During maturation of DCs, Ag-loaded DCs migrate spontaneously to secondary lymph nodes and acquire the capacity to stimulate T cells. These DCs produce pro-inflammatory cytokines that determine or crucially influence the induction of specific types of cytokine-producing CD4 helper T cells and CD8 cytotoxic T cells [9,14]. However, the outcome of T cell stimulation by DCs can also be apoptosis depending on the state of maturation of the DCs [15,16].

Sulfated polysaccharides possess certain biological activities such as anti-virus, anti-tumor, anti-inflammatory and anti-coagulant activities [17,18,19]. Moreover, fucoidan, a sulfated polysaccharide, has been well defined as a maturation-inducing agent on human monocyte-derived DCs (MDDCs), peripheral blood DCs (PBDCs) and mouse bone marrow-derived DCs (BMDCs) [20,21,22]. However sulfated polysaccharide-mediated immune responses *in vivo*, especially DC maturation and T cell activation, have not been fully investigated. The present study was undertaken to test the hypothesis that *in vivo* administration of ascophyllan, a sulfated polysaccharide from *A. nodosum*, can induce the maturation of DCs and the consequent activation of T cell responses. Moreover, we compared the ascophyllan and fucoidan in the effect of DC maturation *in vivo* and *in vitro*. These results may provide a potential new therapeutic strategy to combat viral and bacterial infection and cancer. 

## 2. Results and Discussion

### 2.1. Ascophyllan Promotes the Activation of BMDCs

**Figure 1:**
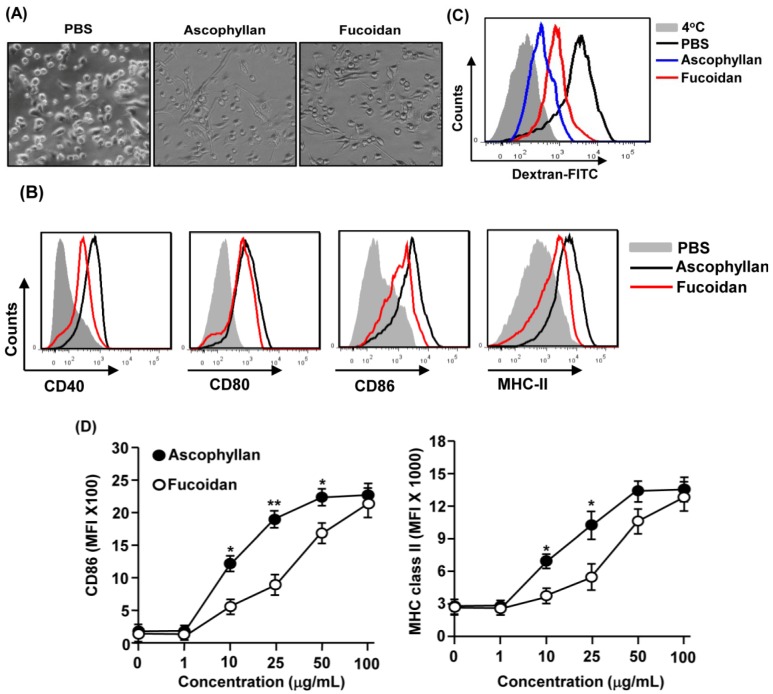
Activation of bone marrow-derived dendritic cells (BMDCs) by ascophyllan. Bone marrow cells (1 × 10^6^) were incubated with 20 ng/mL granulocyte-macrophage colony-stimulating factor (GM-CSF) and 20 ng/mL interleukin-4 (IL-4) for six days, and then stimulated with ascophyllan or fucoidan for 24 h. (**A**) Morphological changes were analyzed by microscopy; (**B**) Expression of surface co-stimulatory molecules measured by flow cytometry; (**C**) Dextran uptake was analyzed from ascophyllan- or fucoidan-treated BMDCs after incubation with dextran-FITC for 1 h at 4 or 37 *°*C; (**D**) CD86 and MHC class II expression levels were measured in ascophyllan- or fucoidan-treated BMDCs at indicated dose (* *p* < 0.05, ** *p* < 0.01 compared to fucoidan). All data are representative of or the average of analyses of six independent samples.

Previous reports showed that ascophyllan can induce activation of mouse macrophages. We therefore assessed whether ascophyllan can also induce activation or maturation of mouse BMDCs *in vitro*. Bone marrow cells were isolated from C57BL/6 mice and cultured with granulocyte-macrophage colony-stimulating factor (GM-CSF) and interleukin-4 (IL-4) to generate immature BMDCs. After six days of culture, the majority of the cells were immature BMDCs based on the expression of CD11c. We further stimulated these cells with 50 μg/mL ascophyllan or with 50 μg/mL fucoidan as a positive control. After 24 h of culture, we noticed that ascophyllan treatment promoted the dendritic morphological changes in BMDCs (Figure 1A). Moreover, the expression levels of co-stimulatory molecules CD40, CD80, CD86 and MHC class II on BMDCs were markedly increased by ascophyllan (Figure 1B). Next, we measured phagocytic activity, which is a specific function of immature DCs. The results revealed that the phagocytic activity of BMDCs had undergone a notable decrease after ascophyllan treatment (Figure 1C). Interestingly, ascophyllan treatment showed a stronger effect on inducing BMDC activation than fucoidan as indicated by higher levels of CD40, CD86 and MHC class II and lower levels of dextran uptake in the BMDCs (Figure 1B,C). To confirm this observation, we assessed the dose-dependent effect of ascophyllan and fucoidan on BMDC activation. As shown in Figure 1D, ascophyllan treatment at 10 or 25 μg/mL induced more potent up-regulation of CD86 and MHC class II compared to fucoidan at the same doses. These data demonstrate that ascophyllan induces activation of BMDCs and its effect is stronger than that of fucoidan.

### 2.2. Ascophyllan Induces the Activation of Spleen DCs in Vivo

**Figure 2:**
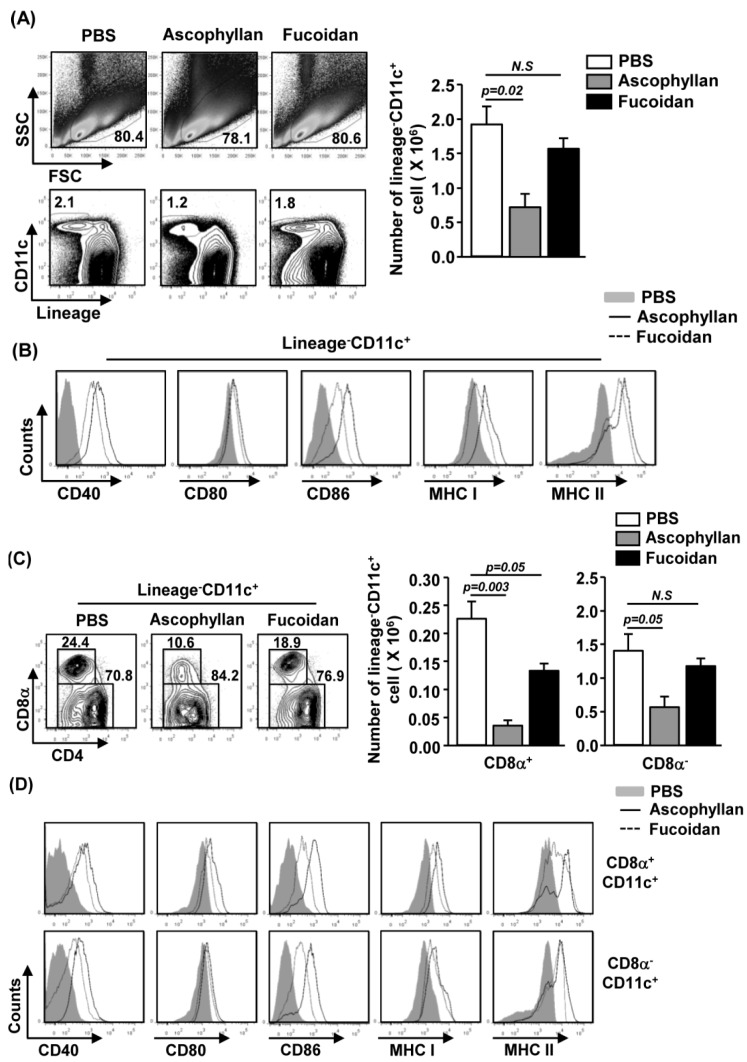
*In vivo* administration of ascophyllan induces spleen conventional DC (cDC) activation. C57BL/6 mice were injected intravenously (*i.v.*) with 50 mg/kg ascophyllan or 50 mg/kg fucoidan for 24 h. (**A**) Percentage of lineage^−^CD11c^+^ cDCs was analyzed by flow cytometry (left panels). Absolute numbers of live, lineage^−^CD11c^+^ cells were shown (right panel); (**B**) Expression levels of CD40, CD80, CD86, MHC class I and MHC class II were measured by flow cytometry; (**C**) Percentage of lineage^−^CD11c^+^CD8α^+^ or lineage^−^CD11c^+^CD8α^−^ cDCs was analyzed on a flow cytometry (left panels). Absolute numbers of live, lineage^−^CD11c^+^CD8α^+^ cells (middle panel) or lineage^−^CD11c^+^CD8α^−^ cells (right panel) were shown (right panel); (**D**) Expression level of CD40, CD80, CD86, MHC class I and MHC class II were measured by flow cytometry. All data are representative of or the average of analyses of six independent samples.

Our *in vitro* observation that ascophyllan promotes BMDC activation prompted us to investigate the effect of ascophyllan on spleen DC activation *in vivo*. We injected 50 mg/kg ascophyllan intravenously (*i.v.*) to C57BL/6 mice and analyzed spleen DCs 24 h later. Ascophyllan treatment led to a significant decrease in the proportion and number of spleen cDCs, which were identified as lineage^−^CD11c^+^ cells, whereas fucoidan had no significant effect (Figure 2A, *p* = 0.02). Moreover, ascophyllan administration induced a substantial increase in the surface levels of CD40, CD80, CD86, MHC class I and MHC class II in spleen cDCs (Figure 2B). 

We next examined the effect of ascophyllan on the expression of co-stimulatory molecules in CD8α^+^ and CD8α^−^ cDC sub-populations 24 h after injection of ascophyllan or fucoidan. As shown in Figure 2C, ascophyllan treatment led to a marked decrease in CD8α^+^ and CD8α^−^ cDC numbers, whereas fucoidan treatment induced a decrease in the number of CD8α^+^ cDCs without affecting the CD8α^−^ cDCs. Furthermore, the expression levels of CD40, CD80, CD86, MHC class I and MHC class II were markedly increased on both CD8α^+^ and CD8α^−^ cDCs by ascophyllan treatment (Figure 2D). Consistent with the results obtained from BMDCs, ascophyllan induced a greater up-regulation of CD40, CD80, CD86, MHC class I and II in spleen CD8α^+^CD11c^+^ cDCs and a greater up-regulation of CD40 and CD86 in spleen CD8α^−^CD11c^+^ cDCs *in vivo* compared to fucoidan. These data indicate that systemic administration of ascophyllan induces activation of spleen cDC *in vivo* and its effect is stronger than fucoidan.

### 2.3. Ascophyllan Promotes Pro-Inflammatory Cytokine Production from DCs

**Figure 3:**
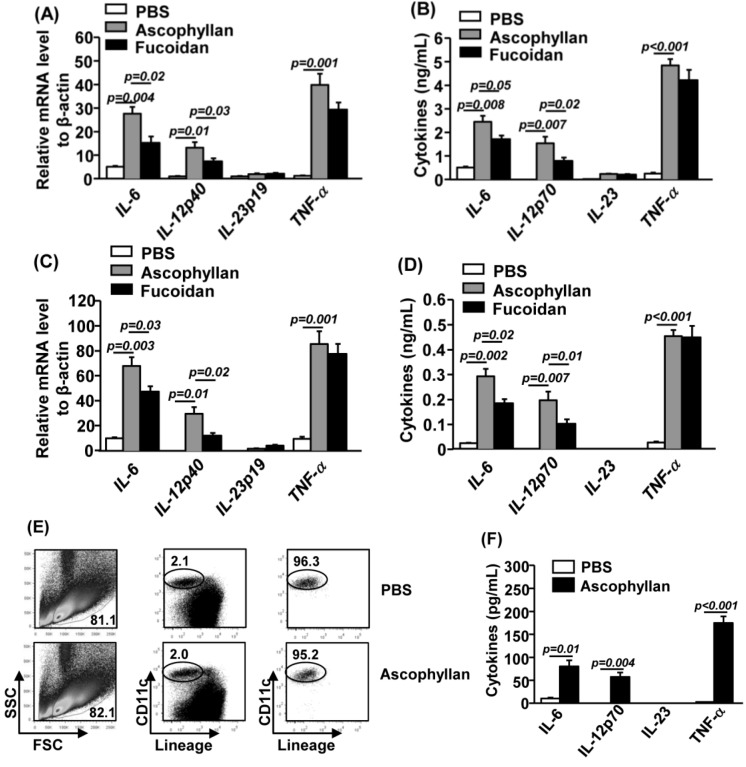
Ascophyllan induces pro-inflammatory cytokine production. BMDC were incubated with 50 μg/mL ascophyllan or fucoidan for 2 h or 24 h. (**A**) mRNA levels of IL-6, IL-12p40, IL-23p19 and TNF-α in BMDCs were measured 2 h after treatment; (**B**) IL-6, IL-12p70, IL-23 and TNF-α levels in culture supernatant from ascophyllan- or fucoidan-treated BMCDs 24 h after treatment; (**C**) Cytokine mRNA levels in splenocytes were measured 2 h after 50 mg/kg ascophyllan or 50 mg/kg fucoidan injection; (**D**) Cytokine concentrations in sera from ascophyllan- or fucoidan-treated mice are shown; (**E**) Lineage^−^CD11c^+^ cDCs were electronically sorted on a FACS Aria II (Becton Bicknson) 2 h after ascophyllan injection. The purities of the sorted cells are shown; (**F**) Purified cDCs were incubated in culture medium for 4 h, and cytokine concentrations in the culture medium were measured by ELISA. All data are representative or the average of analyses of at least six samples for each group.

Matured DCs produce high levels of pro-inflammatory cytokines. To determine whether ascophyllan affects production of cytokines, we treated BMDCs or spleen DCs with ascophyllan for 2 h or 24 h and analyzed the production of pro-inflammatory cytokines. Ascophyllan treatment of BMDCs for 2 h induced up-regulation of IL-6, IL-12p40 and TNF-α mRNA levels but not IL-23p19 (Figure 3A). The secretion levels of IL-6, IL-12p70 and TNF-α were also dramatically increased in cultured medium of BMDCs treated with ascophyllan for 24 h (Figure 3B). Moreover, *in vivo* administration of ascophyllan to C57BL/6 mice caused a marked increase in mRNA levels of IL-6, IL-12p40 and TNF-α from splenocytes 2 h post-injection compared to PBS-treated control mice (Figure 3C). Serum levels of IL-6, IL-12p70 and TNF-α were also substantially increased by ascophyllan (Figure 3D). Consistent with its effect on co-stimulatory molecule expression, ascophyllan induced greater up-regulation of IL-6 and IL-12, but not TNF-α than fucoidan. Interestingly, ascophyllan and fucoidan did not induce IL-23 production in either BMDCs *in vitro* or splenocytes *in vivo* (Figure 3B,D). 

To specifically measure the production of cytokines by spleen cDCs, we isolated lineage^−^CD11c^+^ cDCs from splenocytes by electronic sorting 2 h after ascophyllan administration, and incubated these cells in culture medium for 4 h (Figure 3E). Purified CD11c^+^ cDCs from mice treated with ascophyllan secreted dramatically higher amounts of IL-6, IL-12p70 and TNF-α than those from PBS-treated mice (Figure 3F). Therefore, systemic administration of ascophyllan induced maturation of spleen cDCs as indicated by up-regulation of co-stimulatory molecules and production of pro-inflammatory cytokines. 

Ascophyllan is a fucan, and it has structural differences with fucoidan based on a backbone of uronic acid (mannuronic acid) with fucose containing braches (3-*O*-d-xylosyl-l-fucose-4-sulfate) [3,4]. Although fucoidan and ascophyllan have a number of structural and bioactive similarities, they also possess different functions. Ascophyllan promotes growth of normal kidney MDCK cells, whereas fucoidan is toxic to these cells [23]. Moreover, ascophyllan can induce much higher levels of NO production in RAW264.7 cells than fucoidan [5]. Although, fucoidan has been well defined as a maturation-inducing agent on human and mouse DCs *in vitro* [20,21,22], we demonstrate that ascophyllan can also promote DC maturation, both *in vitro* and *in vivo*, and its effect is stronger than fucoidan. These results are in line with the previous study performed using RAW264.7 cells *in vitro*. 

### 2.4. Ascophyllan Promotes Generation of Th1 and Tc1 Cells in Vivo

**Figure 4:**
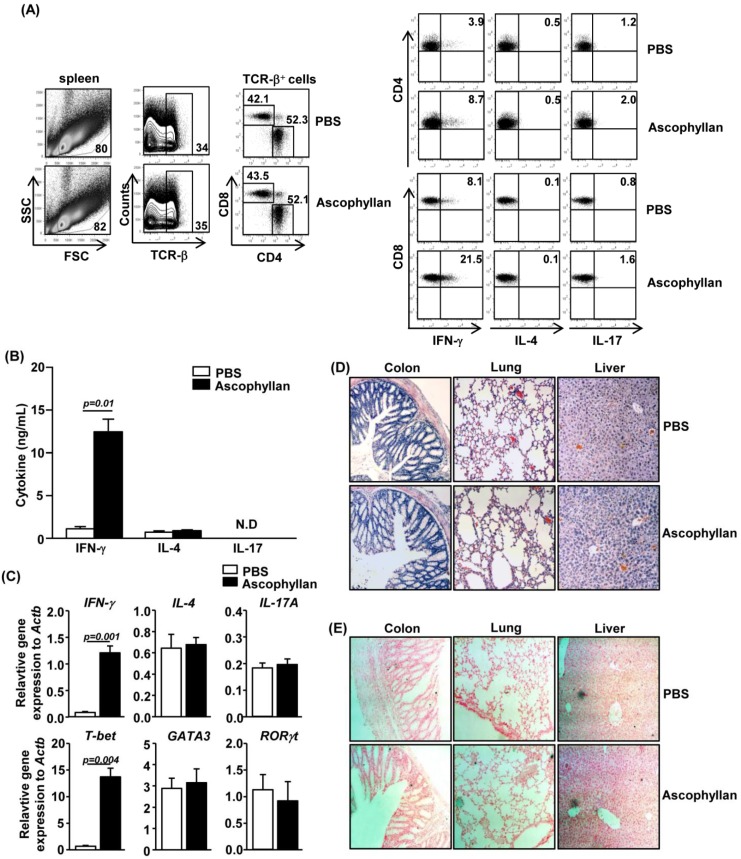
Ascophyllan promotes IFN-γ production in CD4^+^ and CD8^+^ T cells *in vivo*. C57BL/6 mice were injected *i.v.* with 50 mg/kg ascophyllan and 3 days later, injected again with the same amount of ascophyllan. Analyses were done 3 days after the second injection. (**A**) Percentage of IFN-γ-, IL-4- or IL-17-positive cells within CD4^+^ (right upper panels) and CD8^+^ T (right lower panels) cells in spleen were assessed by flow cytometric analysis; (**B**) IFN-γ, IL-4 and IL-17 concentrations in sera were measured by ELISA; (**C**) Cytokine mRNA levels in spleen were measured 24 h after ascophyllan injection; (**D**) Hematoxylin and eosin staining of colon, lung and liver sections; (**E**) *In situ* detection of cell apoptosis in colon, lung and liver. Arrows indicated the apoptotic cells. All data are representative of six samples from three independent experiments.

Cytokine-producing DCs play a key role in the generation of effector T cell subsets, including Th1, Th2 and Th17 cells. Since ascophyllan can induce the maturation of CD8α^+^ and CD8α^−^ cDCs, we next assessed whether ascophyllan-induced mature spleen cDCs can in turn promote CD4^+^ and CD8^+^ T cell responses in an *in vivo* setting. C56BL/6 mice received *i.v.* injection of 50 mg/kg ascophyllan twice, 3 days apart, and were analyzed 3 days after the second injection. Ascophyllan treatment led to marked increases in the proportions of CD4^+^ and CD8^+^ T cells in the spleen that produced IFN-γ, the critical cytokines for Th1 and Tc1 cells, whereas it did not significantly increase the percentages of IL-17- or IL-4-producing CD4^+^ and CD8^+^ T cells (Figure 4A). Moreover, serum levels of IFN-γ were also markedly increased by ascophyllan treatment (Figure 4B). Furthermore, ascophyllan injection induced a marked increase in the expression of IFN-γ and T-bet mRNA, the critical transcription factor for Th1 and Tc1 cells, in the spleen 24 h after injection. However, the mRNA levels of GATA3 and RORγt, signature transcription factors for Th2 and Th17, were not altered by ascophyllan treatment (Figure 4C). To determine whether ascophyllan-induced Th1 and Tc1 responses result in tissue damages or leukocyte infiltration in major organs including colon, lung and liver, we performed H&E staining and *in situ* TUNEL assay on sections of these organs. There was no leukocyte infiltration in colon, lung and liver in either control mice or ascophyllan-treated mice (Figure 4D) and there was no increase in apoptosis in these organs in ascophyllan-treated mice compared to control mice (Figure 4E). These data indicate that ascophyllan enhances Th1 and Tc1 responses without tissue damage *in vivo*.

Different subsets of DCs have different abilities and modes to present antigens and stimulate T cells [9,24]. CD8α^+^ cDCs can efficiently cross-present exogenous soluble and cell-bound antigens through MHC class I, and as a result induce CD8^+^ cytotoxic T cell (CTL) activation [11]. In contrast, CD8α^−^ cDCs present extracellular exogenous antigens through MHC class II, and promote CD4^+^ helper T cell activation [13]. Because CD8α^+^ cDCs are highly specialized in cross-priming CTL response, they have been the main targets in strategies aiming at enhancing anti-virus and anti-tumor responses. In this study, we demonstrate that systemic administration of ascophyllan induces maturation of both CD8α^+^ and CD8α^−^ cDCs. These findings suggest that ascophyllan has the potential ability to enhance not only CD8α^−^ cDCs-mediated direct presentation but also CD8α^+^ cDCs-mediated cross-presentation of antigens. Although ascophyllan induces DC maturation *in vivo*, further research is required to determine whether ascophyllan can promote presentation of cancer or viral Ags by DCs as an effective adjuvant. 

Interestingly, we found that spleen cDC number is substantially decreased by ascophyllan treatment. It has been shown that as a result of T cell activation induced by fully matured DCs, the DCs can undergo apoptosis or anergy [15,16]. Therefore, we examined whether ascophyllan-induced decrease in the number of spleen DCs may result from increased apoptosis. The percentage of spleen cDCs that were undergoing apoptosis, as indicated by positive staining by Annexin V, was significantly increased by ascophyllan treatment (Supplementary Figure S1A). Moreover, the numbers of DCs in mesenteric lymph nodes (mLNs), lung and liver were not altered by ascophyllan treatment, suggesting that there is no significant migration of spleen cDCs into peripheral tissues (Supplementary Figure S1B,C). Thus, ascophyllan-induced decrease in spleen cDC numbers likely result from an increased apoptosis of these cells but not from the migration of these cells to lymph nodes or other organs. In summary, ascophyllan treatment induces full maturation of spleen cDCs that in turn stimulate T cells, which can happen rapidly within 24 h of ascophyllan treatment. Consistent with this, we found that ascophyllan indeed promotes Th1 and Tc1 immune responses, which is indicated by increased IFN-γ and T-bet expression within 24 h treatment of ascophyllan.

### 2.5. Ascophyllan-Induced Generation of Th1 and Tc1 is Dependent on IL-12

**Figure 5:**
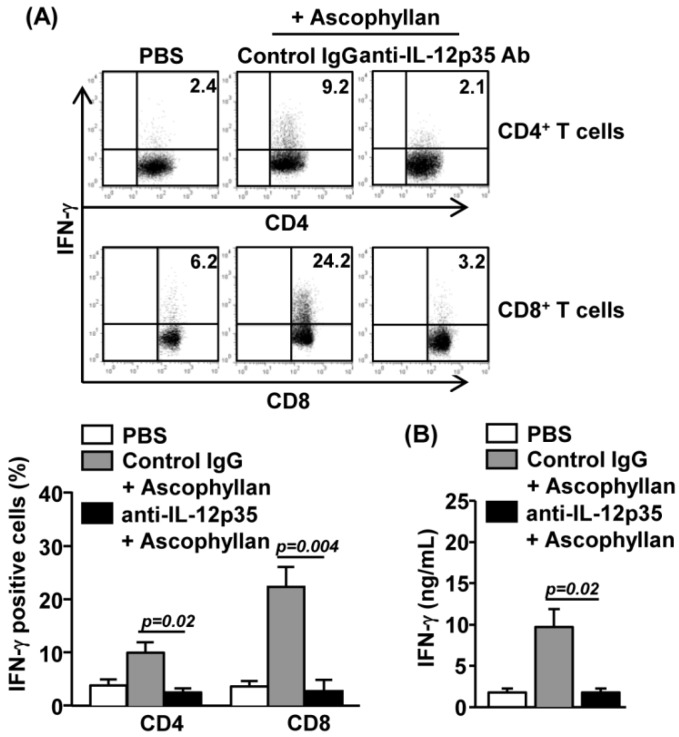
Ascophyllan induces IFN-γ-producing T cells in an IL-12-dependent manner. Anti-IL-12p35 or control IgG Ab were injected intraperitoneally (*i.p.*) to C57BL/6 mice that had received prior *i.v.* injection of ascophyllan. (**A**) Intracellular IFN-γ expressions in CD4^+^ or CD8^+^ T cells were analyzed by flow cytometry (upper panel) and mean percentage of IFN-γ-positive cells were shown (lower panel); (**B**) IFN-γ production levels in sera were measured by ELISA. All data are representative of six samples from three independent experiments.

We next examined whether ascophyllan-induced enhancement of Th1 and Tc1 responses is dependent on IL-12, a dominant inducer of Th1 and Tc1 cells in various immune responses. We injected intraperitoneally (*i.p.*) a neutralizing anti-IL-12p35 Ab into C57BL/6 mice that had received prior *i.v.* injection of ascophyllan. The promoting effect of IFN-γ production in CD4*^+^* and CD8^+^ T cells by ascophyllan was almost completely abrogated by IL-12p35 neutralization (Figure 5A). Moreover, serum IFN-γ levels in mice that received ascophyllan injection were markedly diminished by anti-IL-12p35 treatment. Hence, ascophyllan promotes the generation of IFN-γ-producing Th1 and Tc1 cells in an IL-12-dependent manner (Figure 5B). Together with the observation that ascophyllan enhances IL-12 production by cDCs, these data suggest that ascophyllan promotes Th1 and Tc1 responses by enhancing IL-12 production.

### 2.6. MyD88 Signaling Pathway is Essential for Ascophyllan-Induced DC Maturation

**Figure 6:**
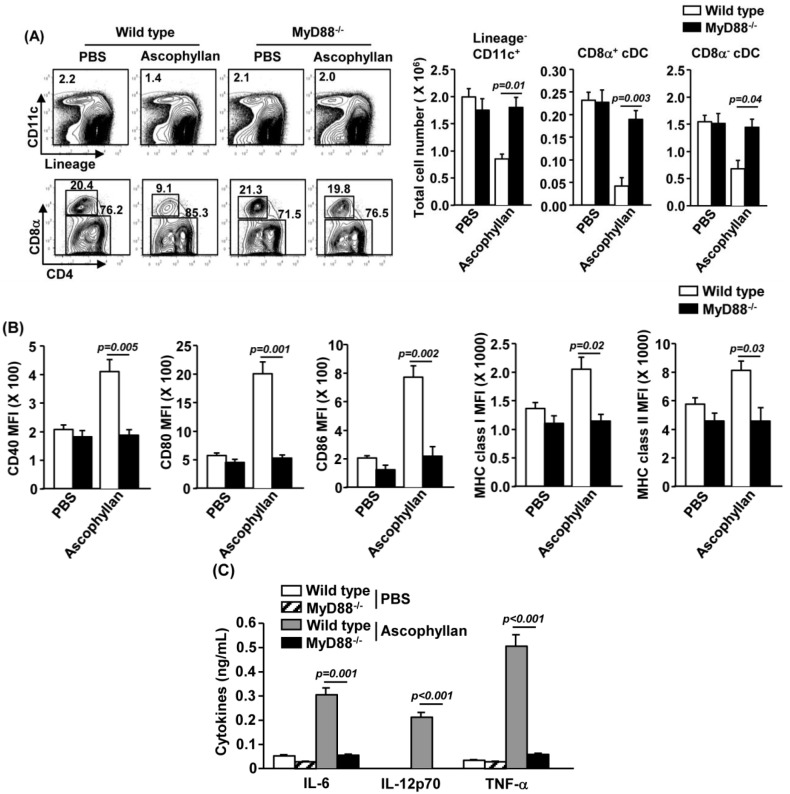
Myeloid differentiation primary response 88 (MyD88) signaling pathway is essential for ascophyllan-induced DC maturation. C57BL/6 or MyD88^−/−^ mice received *i.v.* injection of phosphate buffered saline (PBS) or 50 mg/kg ascophyllan and were analyzed 24 h later. (**A**) Percentage of lineage^−^CD11c^+^ cDCs and CD8α^+^ and CD8α^−^ cDCs was analyzed by flow cytometry (left panels). Absolute numbers of spleen cDCs and cDC subsets were shown (right panels); (**B**) Expression levels of CD40, CD80, CD86, MHC class I and MHC class II were measured by flow cytometry; (**C**) IL-6, IL-12p70 and TNF-α concentrations in sera were shown. All data are representative or the average of analyses of six samples for each group.

To further elucidate the mechanism by which ascophyllan promotes maturation of DCs, we examined the activation of MyD88 signaling pathway, a critical adaptor protein in innate immunity signal transduction by TLR stimulation [25]. C57BL/6 or MyD88**^−^**^/−^ mice were injected *i.v.* with PBS or ascophyllan. As shown in Figure 6A, ascophyllan-induced decrease in lineage^−^CD11c^+^ cDC, CD8α^+^ or CD8α^−^ cDC numbers was almost completely abrogated by MyD88-deficieny. Moreover, ascophyllan-induced up-regulation of CD40, CD80, CD86, MHC class I and MHC class II was also almost completely abolished by MyD88-deficiency (Figure 6B). In addition, ascophyllan-induced production of IL-6, IL-12 and TNF-α was also severely impaired in MyD88^−/−^ mice (Figure 6C). Taken together, these results suggest that MyD88 signaling pathway is essential for ascophyllan-induced DC maturation.

DCs can directly sense pathogen components by pattern recognition receptors (PRRs), such as toll like receptors (TLRs), scavenger receptors (SRs), C-type lectins, mannose receptors and complement receptors [26]. Recently, studies have shown that various types of polysaccharides can activate DCs by combining with the TLRs [27,28,29]. Moreover, many lines of research showed that MyD88 signaling pathway is essential for DC maturation mediated by TLR ligands [27,30,31]. Consistent with these findings, we also showed that ascophyllan cannot promote maturation of spleen cDCs in MyD88^−/−^ mice. These data suggest that ascophyllan may stimulate TLRs to promote spleen cDC maturation. To test this possibility, our future studies will investigate whether ascophyllan can induce DC maturation in TLR ablated mice. Moreover, we will define the signaling pathways activated by ascophyllan in spleen cDCs by protein analysis.

## 3. Experimental Section

### 3.1. Mice

C57BL/6 and MyD88-knock out (MyD88^−/−^) mice were purchased from Shanghai Public Health Clinical Center, and kept under pathogen-free conditions. All experiments were carried out under the guidelines of the Institutional Animal Care and Use Committee at the Shanghai Public Health Clinical Center. The protocol was approved by the committee on the Ethics of Animal Experiments of the Shanghai Public Health Clinical Center (Animal Protocol Number: SYXK-2010-0098).

### 3.2. Chemicals and Cytokines

Ascophyllan was prepared from the powdered *A. nodosum* as described previously [3,5]. Ascophyllan solution was passed through an endotoxin-removal column (Detoxi-gel: Thermo Fisher Scientific, Waltham, MA, USA), and subsequently filtered through an endotoxin-removal filter (Zetapor Dispo, Wako, Japan). Fucoidan of *focus vesiculosus* was obtained from Sigma-Aldrich. The endotoxin levels in purified ascophyllan were evaluated using a Limulus amebocyte lysate (LAL) assay kit (Lonza, Basel, Switzerland). Ascophyllan and fucoidan used in all experiments contained less than 0.1 endotoxin unit/mL. rmGM-CSF and rmIL-4 were obtained from Peprotech. 

### 3.3. Antibodies

Isotype control antibodies (Abs) (IgG1 or IgG2b), CD11c-APCcy7 or PEcy7 (HL3), CD4-Pacific blue (GK1.5), CD8α-PerCPcy5.5 (YTS169.4), CD40-Alexa Flour-647 (HM40-3), CD80-PE (16-10A1), CD86-PEcy7 (GL-1), anti-MHC class I-APC (AF6-88.5.3), anti-MHC class II-PE (M5/114.15.2) and anti-IL-4-PE (11B11) were from Biolegend (San Diego, CA, USA); anti-IFN-γ-Alexa Fluor-488 (XMG1.2) and anti-IL-17-Pacific blue (TCC11-18H10.1) were from eBioscience (San Diego, CA, USA); and neutralizing Abs against mouse anti-IL-12p35 (C18.2) was purchased from eBioscience (San Diego, CA, USA).

### 3.4. Flow Cytometry Analysis

Cells were washed with phosphate buffered saline (PBS) containing 0.5% BSA, pre-incubated for 15 min with unlabeled isotype control Abs, and then labeled with either fluorescence-conjugated Abs by incubation on ice for 30 min followed by washing with PBS. Cellular debris was then eliminated from the analysis using a gate on forward and side scatter. A viability gate was set using 7 aminoactinomycin D (7AAD) staining. The 7AAD-negative population was subsequently analyzed using a FACS Aria II (Becton Dickinson, Franklin Lakes, NJ, USA) and analyzed using the software FlowJo 8.6 (Tree Star Inc., Ashland, OR, USA). As a control for nonspecific staining, isotype-matched irrelevant mAbs were used. For the detection of DC activation, we gated on mononuclear cells based on forward scatter and side scatter and then further gated on lineage**^−^**CD11c^+^ cells, defined as cDCs. The expression of co-stimulatory molecules was measured on total cDCs, CD8α^+^CD11c^+^ or CD8α**^−^**CD11c^+^ cDCs. For the detection of cytokine producing-T cells, we gated on TCR-β^+^ cells in the mononuclear cells to identify T cells and then further analyzed the intracellular levels of IFN-γ, IL-4 and IL-17 in CD4^+^ or CD8^+^ T cells. 

### 3.5. In Vitro BMDC Generation

The initial cultures were prepared as described elsewhere [32]. Bone marrow nucleated cells (1 × 10^6^ cells/mL) were cultured in 5 mL modified RPMI 1640 medium containing 10% FBS in 6 well plates. 20 ng/mL rmGM-CSF plus 20 ng/mL rmIL-4 were added in the medium to support the generation of BMDCs. Unless otherwise stated, cells were cultured for 6 days at 37 °C under 10% CO_2_. The cultured cells were washed twice in fresh medium before additional experiments.

### 3.6. Spleen cDC Analysis

Spleen DCs were analyzed as described elsewhere [33]. Briefly, spleens were cut into small fragments and digested, with 2% fetal bovine serum (FCS) containing collagenase for 20 min at room temperature. Cells from the digest were centrifuged to a pellet and the pellet was re-suspended in 5 mL of a 1.077 histopaque (Sigma-Aldrich, St. Louis, MO, USA). Additional histopaque was layered below and EDTA-FCS was layered above the cell suspension, which was then centrifuged at 1700 g for 10 min. The light density fraction (<1.077 g/cm^3^) was collected and incubated for 30 min with the following FITC-conjugated monoclonal antibodies (mAbs): anti-CD3 (17A2), anti-Thy1.1 (OX-7), anti-B220 (RA3-6B2), anti-Gr1 (RB68C5), anti-CD49b (DX5) and anti-TER-119 (TER-119). The lineage**^−^**CD11c^+^ cells were defined as cDCs, which were further divided into CD8α^+^ and CD8α**^−^** cDCs. Analysis was carried out on a FACS Aria II (Becton Dickinson, Franklin Lakes, NJ, USA).

### 3.7. Apoptosis Assay

Spleen cDCs were analyzed for apoptosis by Annexin V staining kit (Biolegend, San Diego, CA, USA) according to the manufacturer’s instruction. Briefly, splenocytes were stained with anti-lineage and anti-CD11c Abs before they were stained with Annexin V for 15 min. Apoptosis of lineage^−^CD11c^+^ cDCs was then analyzed on a FACS Aria II (Becton Dickinson, Franklin Lakes, NJ, USA). 

### 3.8. Ex Vivo T Cell Stimulation and Intracellular Cytokine Staining

Cells prepared from spleen were stimulated *in vitro* for 4 h with phorbol 12-myristate 13-acetate (50 ng/mL) and ionomycin (1 μM; both from Calbiochem, Darmstadt, Germany), with the addition of monensin solution (Biolegend, San Diego, CA, USA) during the final 2 h. Cells were then stained for surface markers. For intracellular cytokine staining, cells were stained for surface molecules first, then fixed and permeabilized with Cytofix/Cytoperm buffer (eBioscience, San Diego, CA, USA) before incubation with the indicated anti-cytokine Abs in Perm/Wash buffer (eBioscience, San Diego, CA, USA) for 30 min. Control staining with isotype control IgGs was performed in all the experiments. 

### 3.9. ELISA Assay

IL-6, IL-12p70, IL-23(p19/p40) and TNF-α concentrations in the sera or cultured medium were measured in triplicate using standard ELISA kits (Biolegend, San Diego, CA, USA), with standard cytokine preparations being used as the internal controls.

### 3.10. Real-Time qPCR

Total RNA was reverse-transcribed into cDNA using Oligo (dT) and M-MLV reverse transcriptase (Promega, Madison, WI, USA). The cDNA was subjected to real-time PCR amplification (Qiagen, Limburg, The Netherlands) for 40 cycles with annealing and extension temperature at 60 °C, on a LightCycler 480 Real-Time PCR System (Roche, Basel, Switzerland). Primer sequences are: mouse β-Actin forward, 5′-TGGATGACGATATCGCTGCG-3′; reverse, 5′-AGGGTCAGGATACCTCTCTT-3′, IL-12p40 forward, 5′-CACATCTGCTGCTCCACAAG-3′; reverse, 5′-CCGTCCGGAGTAATTTGGTG-3′ IL-23p19 forward, 5′-CTCTCGGAATCTCTGCATGC-3′; reverse, 5′-ACCATCTTCACACTGGATACG-3′ T-bet forward, 5′-CAACAACCCCTTTGCCAAAG-3′; reverse, 5′-TCCCCCAAGCATTGACAGT-3′, IFN-γ forward, 5′-GGATGCATTCATGAGTATTGC-3′; reverse, 5′-CTTTTCCGCTTCCTGAGG-3′, IL-17A forward, 5′-GCGCAAAAGTGAGCTCCAGA-3′; reverse 5′-ACAGAGGGATATCTATCAGGG-3′, TNF-α forward, 5′-CCTTTCACTCACTGGCCCAA-3′; reverse, 5′-AGTGCCTCTTCTGCCAGTTC-3′. Primer sequences are available upon request.

### 3.11. Histology and in Situ Apoptosis Detection

Tissue samples were fixed in 4% paraformaldehyde, embedded in paraffin and sectioned to 5 μm thickness from different areas across the tissue. Paraffin embedded tissue sections were de-paraffinized, hydrated and then stained with hematoxylin and eosin (H&E) and examined for the presence of leukocytes under a microscope. Some tissue sections were de-paraffinized, hydrated and then subjected to *in situ* apoptosis assay using TrevigenTACS.XL *In Situ* Apoptosis Detection kit (R&D Systems) according to the manufacturer’s instruction. Briefly, tissue sections were partially digested with proteinase K for 20 min and then incubated in 3% H_2_O_2_ to inactivated endogenous peroxidases. DNA fragmentation was then detected following the manufacturer’s protocol.

### 3.12. Statistical Analysis

Results are expressed as the mean ± standard error of the mean (SEM). The statistical significance of differences between experimental groups was calculated using analysis of variance with a Bonferroni post-test or an unpaired Student’s *t*-test. All *p*-values <0.05 were considered significant.

## 4. Conclusions

In conclusion, in the present study, we demonstrate a critical function of ascophyllan in promoting mouse DC maturation. This knowledge will enable us to further investigate the potential therapeutic function of ascophyllan in models of infectious disease and cancer, in order to comprehensively understand its effects on various immune cells and immune responses and to develop novel therapeutic strategies. 

## Supplementary Files

Supplementary File 1 Supplementary Information (PDF, 234 KB)

## References

[1] Ramberg J.E., Nelson E.D., Sinnott R.A. (2010). Immunomodulatory dietary polysaccharides: A systematic review of the literature. Nutr. J..

[2] Leung M.Y., Liu C., Koon J.C., Fung K.P. (2006). Polysaccharide biological response modifiers. Immunol. Lett..

[3] Nakayasu S., Soegima R., Yamaguchi K., Oda T. (2009). Biological activities of fucose-containing polysaccharide ascophyllan isolated from the brown alga *Ascophyllum nodosum*. Biosci. Biotechnol. Biochem..

[4] Larsen B., Haug A., Painter T. (1970). Sulphated polysaccharides in brown algae. 3. The native state of dfucoidan in *Ascophyllum nodosum* and *Fucus vesiculosus*. Acta Chem. Scand..

[5] Jiang Z., Okimura T., Yamaguchi K., Oda T. (2011). The potent activity of sulfated polysaccharide, ascophyllan, isolated from *Ascophyllum nodosum* to induce nitric oxide and cytokine production from mouse macrophage RAW264.7 cells: Comparison between ascophyllan and fucoidan. Nitric Oxide.

[6] Nakano K., Kim D., Jiang Z., Ueno M., Okimura T., Yamaguchi K., Oda T. (2012). Immunostimulatory activities of the sulfated polysaccharide ascophyllan from *Ascophyllum nodosum* in *in vivo* and *in vitro* systems. Biosci. Biotechnol. Biochem..

[7] Banchereau J., Steinman R.M. (1998). Dendritic cells and the control of immunity. Nature.

[8] Fujii S., Liu K., Smith C., Bonito A.J., Steinman R.M. (2004). The linkage of innate to adaptive immunity via maturing dendritic cells *in vivo* requires CD40 ligation in addition to antigen presentation and CD80/86 costimulation. J. Exp. Med..

[9] Pooley J.L., Heath W.R., Shortman K. (2001). Cutting edge: Intravenous soluble antigen is presented to CD4 T cells by CD8-dendritic cells, but cross-presented to CD8 T cells by CD8+ dendritic cells. J. Immunol..

[10] Schnorrer P., Behrens G.M., Wilson N.S., Pooley J.L., Smith C.M., El-Sukkari D., Davey G., Kupresanin F., Li M., Maraskovsky E. (2006). The dominant role of CD8+ dendritic cells in cross-presentation is not dictated by antigen capture. Proc. Natl. Acad. Sci. USA.

[11] Shortman K., Heath W.R. (2010). The CD8+ dendritic cell subset. Immunol. Rev..

[12] Villadangos J.A., Schnorrer P. (2007). Intrinsic and cooperative antigen-presenting functions of dendritic-cell subsets *in vivo*. Nat. Rev. Immunol..

[13] Watts C. (2004). The exogenous pathway for antigen presentation on major histocompatibility complex class II and CD1 molecules. Nat. Immunol..

[14] Moser M., Murphy K.M. (2000). Dendritic cell regulation of TH1-TH2 development. Nat. Immunol..

[15] Kurts C., Kosaka H., Carbone F.R., Miller J.F., Heath W.R. (1997). Class I-restricted cross-presentation of exogenous self-antigens leads to deletion of autoreactive CD8(+) T cells. J. Exp. Med..

[16] Tan J.K., O’Neill H.C. (2005). Maturation requirements for dendritic cells in T cell stimulation leading to tolerance *versus* immunity. J. Leukoc. Biol..

[17] Fitton J.H. (2011). Therapies from fucoidan; multifunctional marine polymers. Mar. Drugs.

[18] Kwak J.Y. (2014). Fucoidan as a marine anticancer agent in preclinical development. Mar. Drugs.

[19] Cumashi A., Ushakova N.A., Preobrazhenskaya M.E., D’Incecco A., Piccoli A., Totani L., Tinari N., Morozevich G.E., Berman A.E., Bilan M.I. (2007). A comparative study of the anti-inflammatory, anticoagulant, antiangiogenic, and antiadhesive activities of nine different fucoidans from brown seaweeds. Glycobiology.

[20] Kim M.H., Joo H.G. (2008). Immunostimulatory effects of fucoidan on bone marrow-derived dendritic cells. Immunol. Lett..

[21] Yang M., Ma C., Sun J., Shao Q., Gao W., Zhang Y., Li Z., Xie Q., Dong Z., Qu X. (2008). Fucoidan stimulation induces a functional maturation of human monocyte-derived dendritic cells. Int. Immunopharmacol..

[22] Jin J.O., Park H.Y., Xu Q., Park J.I., Zvyagintseva T., Stonik V.A., Kwak J.Y. (2009). Ligand of scavenger receptor class A indirectly induces maturation of human blood dendritic cells via production of tumor necrosis factor-alpha. Blood.

[23] Jiang Z., Okimura T., Yokose T., Yamasaki Y., Yamaguchi K., Oda T. (2010). Effects of sulfated fucan, ascophyllan, from the brown Alga *Ascophyllum nodosum* on various cell lines: A comparative study on ascophyllan and fucoidan. J. Biosci. Bioeng..

[24] Vremec D., Pooley J., Hochrein H., Wu L., Shortman K. (2000). CD4 and CD8 expression by dendritic cell subtypes in mouse thymus and spleen. J. Immunol..

[25] Takeda K., Akira S. (2004). TLR signaling pathways. Semin. Immunol..

[26] Gordon S. (2002). Pattern recognition receptors: Doubling up for the innate immune response. Cell.

[27] Calzas C., Goyette-Desjardins G., Lemire P., Gagnon F., Lachance C., van Calsteren M.R., Segura M. (2013). Group B Streptococcus and Streptococcus suis capsular polysaccharides induce chemokine production by dendritic cells via Toll-like receptor 2- and MyD88-dependent and -independent pathways. Infect. Immun..

[28] Lin C.Y., Lu M.C., Su J.H., Chu C.L., Shiuan D., Weng C.F., Sung P.J., Huang K.J. (2013). Immunomodulatory effect of marine cembrane-type diterpenoids on dendritic cells. Mar. Drugs.

[29] Wallner S., Lutz-Nicoladoni C., Tripp C.H., Gastl G., Baier G., Penninger J.M., Stoitzner P., Wolf D. (2013). The role of the E3 ligase Cbl-B in murine dendritic cells. PLoS One.

[30] Ochi A., Nguyen A.H., Bedrosian A.S., Mushlin H.M., Zarbakhsh S., Barilla R., Zambirinis C.P., Fallon N.C., Rehman A., Pylayeva-Gupta Y. (2012). MyD88 inhibition amplifies dendritic cell capacity to promote pancreatic carcinogenesis via Th2 cells. J. Exp. Med..

[31] Macedo G.C., Magnani D.M., Carvalho N.B., Bruna-Romero O., Gazzinelli R.T., Oliveira S.C. (2008). Central role of MyD88-dependent dendritic cell maturation and proinflammatory cytokine production to control *Brucella abortus* infection. J. Immunol..

[32] Sathe P., Pooley J., Vremec D., Mintern J., Jin J.O., Wu L., Kwak J.Y., Villadangos J.A., Shortman K. (2011). The acquisition of antigen cross-presentation function by newly formed dendritic cells. J. Immunol..

[33] Vremec D., O’Keeffe M., Wilson A., Ferrero I., Koch U., Radtke F., Scott B., Hertzog P., Villadangos J., Shortman K. (2011). Factors determining the spontaneous activation of splenic dendritic cells in culture. Innate Immun..

