# Supplementary material for: Ascophyllan Purified from Ascophyllum nodosum Induces Th1 and Tc1 Immune Responses by Promoting Dendritic Cell Maturation
Source: Mar Drugs. 2014 Jul 14;12(7):4148–64. doi: 10.3390/md12074148 (PMC4113820; doi:10.3390/md12074148)
Supplement: Supplementary File 1 — Supplementary Information (PDF, 234 KB) [file marinedrugs-12-04148-s001.pdf]

Supplementary Information

Figure S1. Effect of ascophyllan in apoptosis and migration of DCs in peripheral tissue. Ascophyllan was injected into mice described in Figure 4. (**A**) Apoptotic cells were measured in spleen; (**B**) Percentage of lineage^{-}CD11c^{+} DCs in mesenteric lymph node (mLN), lung and liver were analyzed by flow cytometry; (**C**) Absolute numbers of tissue DCs are shown. Data are representative of six samples from two independent experiments.

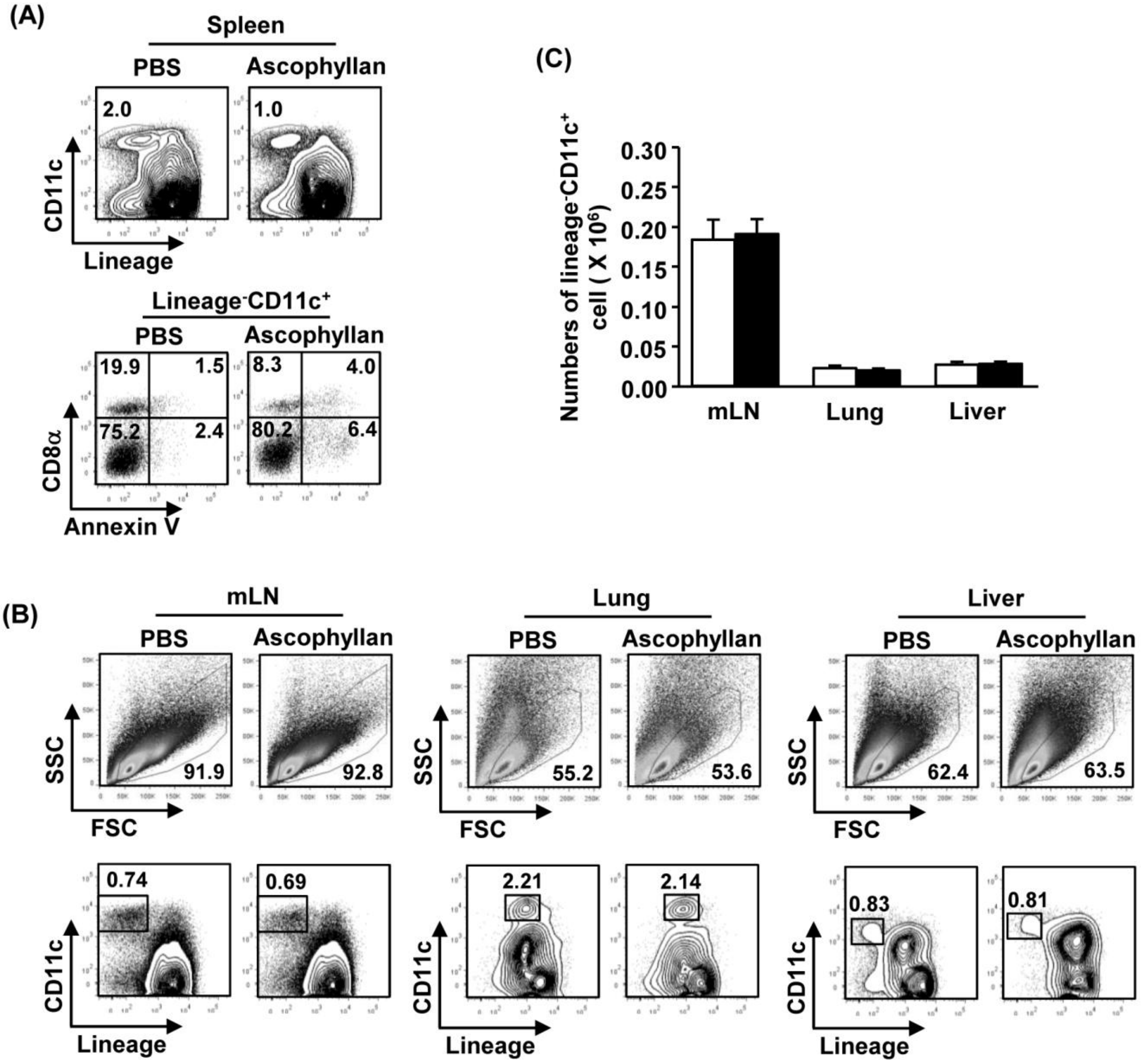

© 2014 by the authors; licensee MDPI, Basel, Switzerland. This article is an open access article distributed under the terms and conditions of the Creative Commons Attribution license (http://creativecommons.org/licenses/by/3.0/).
